# The surgical strategy and technical nuances of *in situ* side-to-side bypass for the management of complex intracranial aneurysms

**DOI:** 10.3389/fneur.2023.1243453

**Published:** 2023-10-17

**Authors:** Hua-wei Wang, Zhe Xue, Cai-hong Sun, Dong-sheng Kong, Chen Wu, Zheng-hui Sun

**Affiliations:** Department of Neurosurgery, The First Medical Center of PLA General Hospital, Beijing, China

**Keywords:** revascularization, intracranial-intracranial bypass, *in situ* bypass, side-to-side anastomosis, complex intracranial aneurysms

## Abstract

**Background:**

Despite continuous advances in microsurgical and endovascular techniques, the treatment of complex aneurysms remains challenging. Aneurysms that are dilemmatic for conventional clipping or endovascular coiling often require bypass as part of a strategy to reduce the risk of ischemic complications. In anatomically favorable sites, the intracranial–intracranial *in situ* bypass may be an appealing choice. This article details the surgical strategies, operative nuances, and clinical outcomes of this technique with a consecutive series in our department.

**Methods:**

A retrospective review of a prospectively maintained neurosurgical patient database was performed to identify all patients treated with side-to-side *in situ* bypass from January 2016 to June 2022. In total, 12 consecutive patients, including 12 aneurysms, were identified and included in the series. The medical records, surgical videos, neuroimaging studies, and follow-up clinic notes were reviewed for every patient.

**Results:**

Of the 12 aneurysms, there were 5 middle cerebral artery aneurysms, 4 anterior cerebral artery aneurysms, and 3 posterior inferior cerebellar artery aneurysms. The morphology of the aneurysms was fusiform in 8 patients and saccular in the remaining 4 patients. There were 3 patients presented with subarachnoid hemorrhage. The treatment modality was simple *in situ* bypass in 8 cases and *in situ* bypass combined with other modalities in 4 cases. Bypass patency was confirmed in all cases by intraoperative micro-doppler probe and (or) infrared indocyanine green (ICG) video angiography intraoperatively and with digital subtraction angiography (DSA) or computed tomography angiography (CTA) postoperatively. None of the patients developed a clinically manifested stroke due to the procedure though a callosomarginal artery was intentionally removed in one patient. The median follow-up period was 16.2 months (6-36). All patients had achieved improved or unchanged modified Rankin scale scores at the final follow-ups.

**Conclusion:**

Cerebral revascularization technique remains an essential skill for the treatment of complex aneurysms. The *in situ* bypass is one of the most effective techniques to revascularize efferent territory when vital artery sacrifice or occlusion is unavoidable. The configuration of *in situ* bypass should be carefully tailored to each case, with consideration of variations in anatomy and pathology of the complex aneurysms.

## Introduction

With continuous advances in endovascular techniques, the treatment paradigm of intracranial aneurysms has shifted to intervention from microsurgery in the past 2 decades (1). However, whatever modality is selected, the management of complex aneurysms remains challenging due to their giant size, wide neck, dolichoectatic morphology, or perforator features. Overall, the reported mortality and morbidity remain relatively high (2–4). A subset of complex aneurysms that are not suitable for standard intervention or clipping may benefit from surgical revascularization, which could ensure sufficient distal blood flow and lower the risk of ischemic complication when the parent artery or distal branch was deliberately occluded as part of the treatment strategy (5).

There have been reports on the increased use of revascularization techniques for complex aneurysms management recently (6) and that equivalent results have been achieved from extracranial–intracranial (EC-IC) and intracranial-to-intracranial (IC-IC) bypasses in terms of clinical and radiological outcomes (5, 7). Both bypass techniques have been used successfully in our center (8). Nevertheless, bypass preference has changed over time with evolving microsurgical techniques and collective experience and creativity. The IC-IC bypasses (or third-generation bypass) are being more preferred recently due to the simple, elegant, and hemodynamic advantages over their EC-IC counterparts (7, 9). The *in situ* side-to-side anastomose technique epitomizes the appealing IC-IC bypasses.

The *in situ* bypass connects parallel and proximate arteries in a side-to-side fashion. In anatomically favorable sites, for example, the longitudinal fissure, Sylvian fissure, ambient cistern, and cisterna magna, it could provide cross-communication blood flow between the anterior cerebral arteries, the middle cerebral artery branches, the superior cerebellar artery and posterior cerebral artery, and the posterior inferior cerebellar arteries, respectively. However, studies reporting this technique for the management of complex intracranial aneurysms are few (10, 11). In the present study, we will summarize our experience of *in situ* bypasses and detail the surgical strategies, operative nuances, and clinical outcomes with a consecutive series in our department.

## Methods

### Patients and data collection

After obtaining approval from the institutional review board, a consecutive series was identified from a prospectively maintained database of bypasses for managing intracranial aneurysms from January 2016 to June 2022. Only the patients with *in situ* bypass were included in the study. Patients' demographic characteristics, radiographic images, operative videos, and medical records including procedure-related complications were reviewed. The neurological outcomes were assessed using the modified Rankin Scale (mRS) upon discharge and at subsequent follow-up visits or by telephone. A neurosurgeon who was not directly involved in the treatment performed the assessments. Bypass patency and aneurysm occlusion were evaluated using angiography at discharge, half a year, and 1 year postoperatively, and then annually. All patients provided written informed consent for database collection and research use.

### Surgical steps and technical nuances

Though the surgical approaches diversify according to the locations of the aneurysms and associated vascular anatomy, the procedure of side-to-side anastomosis during each *in situ* bypass is largely identical with minor differences. In brief, with full exposure, the parallel recipient and donor arteries were circumferentially dissected. Temporary occlusion could be achieved by 2 clips crossing both vessels or four separate mini-clips clamping each vessel proximally and distally. Alternatively, three clips with one clip crossing both vessels proximally and two mini-clips occluding each artery distally were applied. Overall, the configuration of temporary clip placement should approximate the vessels while optimizing visibility.

Then, a rubber dam was placed beneath both vessels to keep them away from the blood background. A continuous suction drain was recommended in such a deep operative field as well. After methylene blue coloring, both arteries were pierced at approximately 9 o'clock and 3 o'clock positions with a fine syringe needle, and then, angled micro-scissors were used to extend the opening on the superior-medial aspect of both arteries. The length of arteriotomies was typically performed to be approximately 2.5 times the diameter of the artery. Special attention should be given to avoid damaging the vessels' posterior wall.

Two nylon threads (9-0 or 10-0) were cut to approximately 50 mm long for easy handling. Both the stay sutures were performed in an outside–inside–outside fashion at both apices of the arteriotomy. Each stay suture was tied off using a square knot, and the tail with the needle was intentionally left long. One tail was used to run the stitch through the posterior wall, and the other through the anterior wall. Some surgeons advocate small loops are created during suturing and then tightened sequentially after suturing is completed to ensure even tension along the entire suture line (12–14), but in our experience, step-by-step tightening during suturing was also practicable. Heparin irrigation was used intermittently in the surgical field during the anastomosis, and systemic heparin was not necessary. No aspirin therapy was required before and postoperatively.

After the suturing was completed, bypass patency was confirmed with an intraoperative micro-doppler probe and (or) infrared indocyanine green (ICG) video angiography. Small leaks could be stopped by covering them with a small piece of Gelfoam (Upjohn, Kalamazoo, MI) and light pressure, whereas large leaks required a stitch repair. Total intravenous anesthesia was used both to induce and maintain general anesthesia. Somatosensory and motor evoked potentials were monitored in all patients and burst suppression was maintained with propofol or barbiturates during clamp time. The mean blood pressure was maintained at 100 mmHg and was raised by 30% above during the clamp time. Conventional computed tomography (CT) scan and digital subtraction angiography (DSA) or computed tomography angiography (CTA) were used as a common postoperative radiological assessment.

## Results

### Patient and aneurysm characteristics

During a 6-year period from January 2016 to June 2022, 758 patients with intracranial aneurysms were treated by microsurgery in our department. The treatment decision was determined by a multidisciplinary team comprising neurovascular surgeons and interventional neuroradiologists. A bypass procedure was performed to re-perfuse the involved territory whenever a parent artery was to be deliberately sacrificed. After screening, there were in total of 86 patients who had undergone various types of revascularization procedures for aneurysm management in the period. Among them, twelve patients who underwent side-to-side anastomosis with or without other bypasses were identified, representing 13.9% of patients with revascularization surgery.

Of the 12 patients with *in-situ* bypasses, the average age was 48.5 years (range, 11–66), and there was a male predominance (58.3%). Three patients presented with subarachnoid hemorrhage, and two patients had recurrent aneurysms after clipping and one after coiling. The other six patients had asymptomatic aneurysms identified during the evaluation of apparently unrelated complaints. The mean mRS of all patients at presentation was 1.3 (range, 0–4). There were 12 aneurysms in total in the 12 patients, including 5 middle cerebral artery (MCA) aneurysms, 4 anterior cerebral artery (ACA) aneurysms, and 3 posterior inferior cerebellar artery (PICA) aneurysms. The mean diameter of these aneurysms was 17.3 mm, ranging from 6.0 to 28.5 mm. The morphology of the aneurysms was fusiform in eight patients and saccular in the remaining four patients. The above demographic and radiological characteristics of the aneurysms are shown in Table 1.

**Table 1:** Demographic and radiological characteristics of the aneurysms.

**Case no**.	**Age (years)**	**Sex**	**Presentation**	**Aneurysm location**	**Morphology**	**Size (cm)**	**Pre-operative mRS score**
1	64	F	SAH; ICH	R A2-A3	Saccular	1.2	4
2	47	F	Headache	AcomA	Saccular	1	1
3	38	F	Dizziness	L p1-V4	Saccular	1.5	1
4	59	M	Recurrent aneurysms after clipping	R M2	Fusiform	2	0
5	11	M	Recurrent aneurysms after Coiling	L M1-M2	Fusiform, Multi-Lobular	2.5	0
6	66	M	SAH	R p2	Fusiform	0.6	3
7	65	F	SAH; Visual deficit	L A1	Fusiform	1.5	3
8	45	M	Accidental	L p1	Fusiform	1.2	0
9	51	M	Headache	R M2	Fusiform	2.2	1
10	57	M	Cerebral infarction	R M2	Fusiform	2.3	2
11	62	M	Accidental	R A1-A2	Saccular	2	0
12	17	F	Recurrent aneurysms after clipping	R M1-M2	Fusiform	2.8	0

mRS, Modified Rankin Scale; M/F, male/female; R/L, right/left; SAH, subarachnoid hemorrhage; ICH, intracranial hematoma; A, segment of anterior cerebral artery; AcomA, anterior communicating artery; M, segment of middle cerebral artery; p, segment of posterior inferior cerebellar artery; V, segment of vertebral artery.

### Clinical features and surgical results

**Figure 1:**
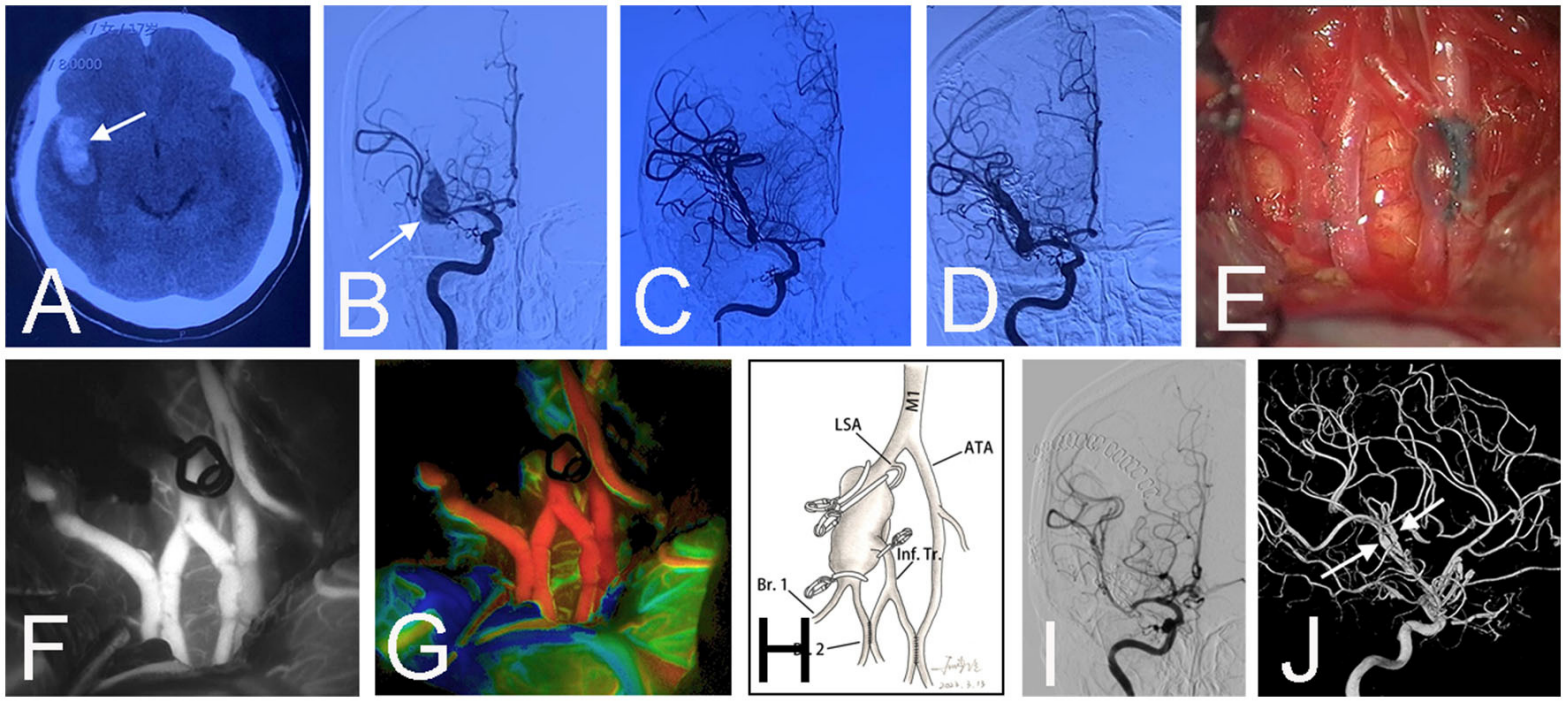
Preoperative CT scan showed a subarachnoid hemorrhage and hematoma above the corpus callosum (arrow) in a 63-year-old female (Case 1) **(A)**. Emergent CT angiography revealed a saccular aneurysm (arrow) at the location where the right anterior cerebral artery branches into the callosomarginal artery and pericallosal artery **(B)**. An emergency surgery was performed at night because the patient had a bad mental state (mRS 4). Surgical clipping was attempted, but the aneurysmal neck was brittle. Two crevasses (arrows) emerged during the aneurysm dissection **(C)**. Then a bail-out side-to-side anastomosis was performed between bilateral pericallosal arteries **(D)**. Intraoperative infrared indocyanine green angiography confirmed the patency of the anastomosis **(E)**. The aneurysm was trapped, and the right callosomarginal artery was sacrificed because it originated from the aneurysmal body and was hard to preserve. Fortunately, no infarction occurred post-surgery **(F)**. The schematic diagram illustrates the treatment in this patient **(G)**. Postoperative angiography demonstrated the obliteration of the aneurysm **(H, I)**. Three-dimensional angiography showed the patent anastomosis (arrow) and right pericallosal artery (arrowhead) **(J)**.

Cranial approaches were chosen individually. Pterional craniotomy was used in all five MCA aneurysms, the far lateral approach in all three PICA aneurysms, the bifrontal craniotomy in three ACA aneurysms, and the bifrontal craniotomy combined with a pterional approach in another ACA aneurysm. The *in situ* bypass was the only revascularization procedure in eight patients and combined with others in four patients. The combined procedures included one M4-superficial temporal artery (STA)-M4 interposition bypass, one M2-M2 reimplantation, one EC-IC STA-M2+M2 double-barrel bypass, and one parallel M2-M2 *in situ* bypass. Aneurysm trapping was performed at one stage in 10 patients, and aneurysm occlusion was achieved by second-stage coiling in 2 patients. The patency of *in situ* bypasses was confirmed in all patients both intraoperatively and postoperatively. The 6-month postoperative radiological assessment demonstrated all the aneurysms were completely obliterated. No mortalities occurred and no technical- or bypass-related morbidities developed though a callosomarginal artery was intentionally removed in one patient (Case 1) (Figure 1). The median follow-up period was 16.2 months (6-36). All patients had improved or unchanged mRS scores at the final follow-up. The clinical features and surgical results of *in situ* side-to-side bypasses are listed in Table 2.

**Table 2:** Clinical features and surgical results of *in situ* side-to-side bypasses.

**Case no**.	**Aneurysm location**	**Cranial approach**	**Bypass technique**	**Aneurysm occlusion**	**Angiography outcome**	**Surgical complication**	**Last mRS score**	**Follow-up (months)**
1	R A2-A3	BAIH	R A3-L A3 *in situ*	Trapping	Patent bypass, complete obliteration of aneurysm	R CmaA Occlusion	1	12
2	Acom	BAIH	R A3-L A3 *in situ*	Second stage coiling	Patent bypass, complete obliteration of aneurysm	No	1	18
3	L p1-V4	Far lateral approach	R p3-L p3 *in situ*	Trapping	Patent bypass, complete obliteration of aneurysm	No	0	36
4	R M2	Pterional approach	R M2-R ATA *in situ*, R M4-STA-M4 interposition	Trapping	Patent bypass, complete obliteration of aneurysm	No	0	8
5	L M1-M2	Pterional approach	L M2-L M2 *in-Situ*, L M2-L M2 reimplantation	Trapping	Patent bypass, complete obliteration of aneurysm	No	0	12
6	R p2	Far lateral approach	R p1-R p3 *in situ*	Trapping	Patent bypass, complete obliteration of aneurysm	No	0	12
7	L A1	BAIH and Left Pterional approach	R A3-L A3 *in situ*	Trapping	Patent bypass, complete obliteration of aneurysm	No	2	18
8	L p1	Far lateral approach	R p3-L p3 *in situ*	Trapping	Patent bypass, complete obliteration of aneurysm	No	0	18
9	R M2	Pterional approach	R M2-R M2 *in situ*, R STA-M2+M2 double barrel	Trapping	Patent bypass, complete obliteration of aneurysm	No	0	24
10	R M2	Pterional approach	R M2-M2 *in situ*	Trapping	Patent bypass, complete obliteration of aneurysm	No	2	18
11	R A1-A2	BAIH	R A3-L A3 *in situ*	Second stage coiling	Patent bypass, complete obliteration of aneurysm	No	0	12
12	R M1-M2	Pterional approach	R M2-R M2+R M2-R M2	Trapping	Patent bypass, complete obliteration of aneurysm	No	0	6

mRS, Modified Rankin Scale; M/F, male/female; R/L, right/left; A, segment of anterior cerebral artery; AcomA, anterior communicating artery; M, segment of middle cerebral artery; p, segment of posterior inferior cerebellar artery; V, segment of vertebral artery; BAIH, basal anterior interhemispheric approach; ATA, anterior temporal artery; STA, superficial temporal artery; CmaA, callosomarginal artery.

## Discussion

This *in situ* side-to-side bypass technique is unique to neurovascular surgery because two neighboring and opposing arteries are seldom observed in vascular structures of other body parts. In 1986, Ikeda et al. (15) first described the microvascular side-to-side anastomosis technique in neurosurgery. After the initial case reports in the early 90s, a few case series discussed the applications of the technique and multiple new construct variations have emerged (9, 10, 14). The *in situ* bypasses are appealing because they are entirely intracranial and less vulnerable to injury, do not require harvesting an extracranial artery or graft, use donor and recipient arteries with diameters that are well matched and require just one anastomosis. Furthermore, the *in situ* bypass configuration forms a communicating artery or vascular bifurcation in a highly anatomically directed fashion, which could minimize the disruption of normal blood flow distal to the aneurysm. The favorable surgical and radiologic outcomes in our series also proved the advantages of *in situ* bypass in the management of complex cerebral aneurysms.

However, *in situ* side-to-side anastomosis is probably the most difficult bypass technique. It is commonly performed in a deep surgical corridor and requires the donor and recipient arteries to lie parallel and in proximity to each other. Fortunately, this uncommon anatomical location spared the need for tedious donor and receipt artery dissection just as required in other type of bypasses. Moreover, side-to-side anastomosis often requires more bites than others because the arteriotomy is long enough to an extent that it is two-to-three times the diameter of the arteries, whereas, with the continuous suturing technique, the amount of time spent could be kept below 45 min, as reported in others' series (14). One potential pitfall while performing the long anastomosis is suturing the two walls of the same artery together so that it gets closed. This could be avoided with an assistant from the intraluminal stent during the anastomosis though it rarely happened with a high magnification view of the microscope and methylene blue coloring of the arterial walls based on our experience.

One criticism toward the *in situ* bypass is that both the donor and recipient vessels have to be clamped to perform the side-to-side anastomosis instead of just temporarily occluding one intracranial recipient artery such as the traditional STA-MCA bypass, which had achieved very good surgical results in reported series (5). Therefore, the bypass failure would jeopardize the patency of two intracranial arteries with a subsequent risk of bilateral or wide-ranging ischemic events. However, this concern did not occur in our series. The results might be attributed to the following reasons. First, the continuous suturing technique decreased the number of knots and the amount of time spent on temporarily occluding. Second, the long arteriotomy promised a high patency rate (100%) of side-to-side anastomosis. Moreover, various methods were used to increase the tolerance of the brain to ischemia during which both arteries were temporarily occluded, including slight hypertension, barbiturates, and mild hypothermia. The electrophysiological monitoring was also very helpful during the temporary occlusive period in our series.

In terms of the management of aneurysms after the *in situ* bypass procedure, radical trapping is recommended. Proximal Hunterian ligation of the parent artery is not promising because the remaining inflow or reverse flow could contribute to aneurysm growth and rupture in spite of intraluminal thrombus formation (16, 17). Furthermore, recurrence or regrowth might occur if there are angiography-negative vessels that reverse flow into the aneurysm. Hauck and Samson (18) reported a subarachnoid hemorrhage occurring after an initial operation, and then during a second operation, they identified several angiography-negative vessels that arose from the aneurysmal dome. Thus, for recurrence-free treatment, aneurysm trapping should be performed whenever possible except for the existence of perforating arteries supplying eloquent regions near the aneurysm, in which partial trapping such as distal or proximal trapping has to be employed. Under the circumstances, the contrast-enhanced ultrasound would be beneficial to study the effect of distal clipping on the aneurysm flow and the parenchymal blood flow after the bypass as reported by Acerbi et al. (19). In certain cases, such as the A1 or anterior communicating aneurysms, simultaneous proximal and distal parent arteries occlusion is hard to achieve in a single craniotomy, and staged endovascular aneurysm occlusion is a better option to simplify the surgical exposure.

Most of the *in situ* bypasses were part of a planned surgical strategy in this series, except in one patient (case 1), in which we initially attempted to perform surgical clipping. Sometimes, the *in situ* bypass is a favorable intraoperative bailout strategy as well. It is particularly attractive in emergent situations when there is an inadvertent vascular injury during an operation or trauma or occlusion of a planned bypass (Figure 1). In this case, furthermore, a double bypass pattern as presented by Acerbi et al. would be more reasonable (20). Moreover, this technique can also be used appropriately as an adjunct to other revascularization procedures. This situation is especially common in MCA aneurysms. The uniquely complex nature of MCA aneurysms, which might not be precisely discerned even using 3-dimensional angiographic reconstructions, can necessitate feasible salvage strategies when intraoperative dissection has revealed unexpected anatomic peculiarities. The *in situ* revascularization will eliminate the added complexities of an unplanned extracranial donor artery dissection or graft harvest associated with EC-IC bypass or IC-IC revascularization.

**Figure 2:**
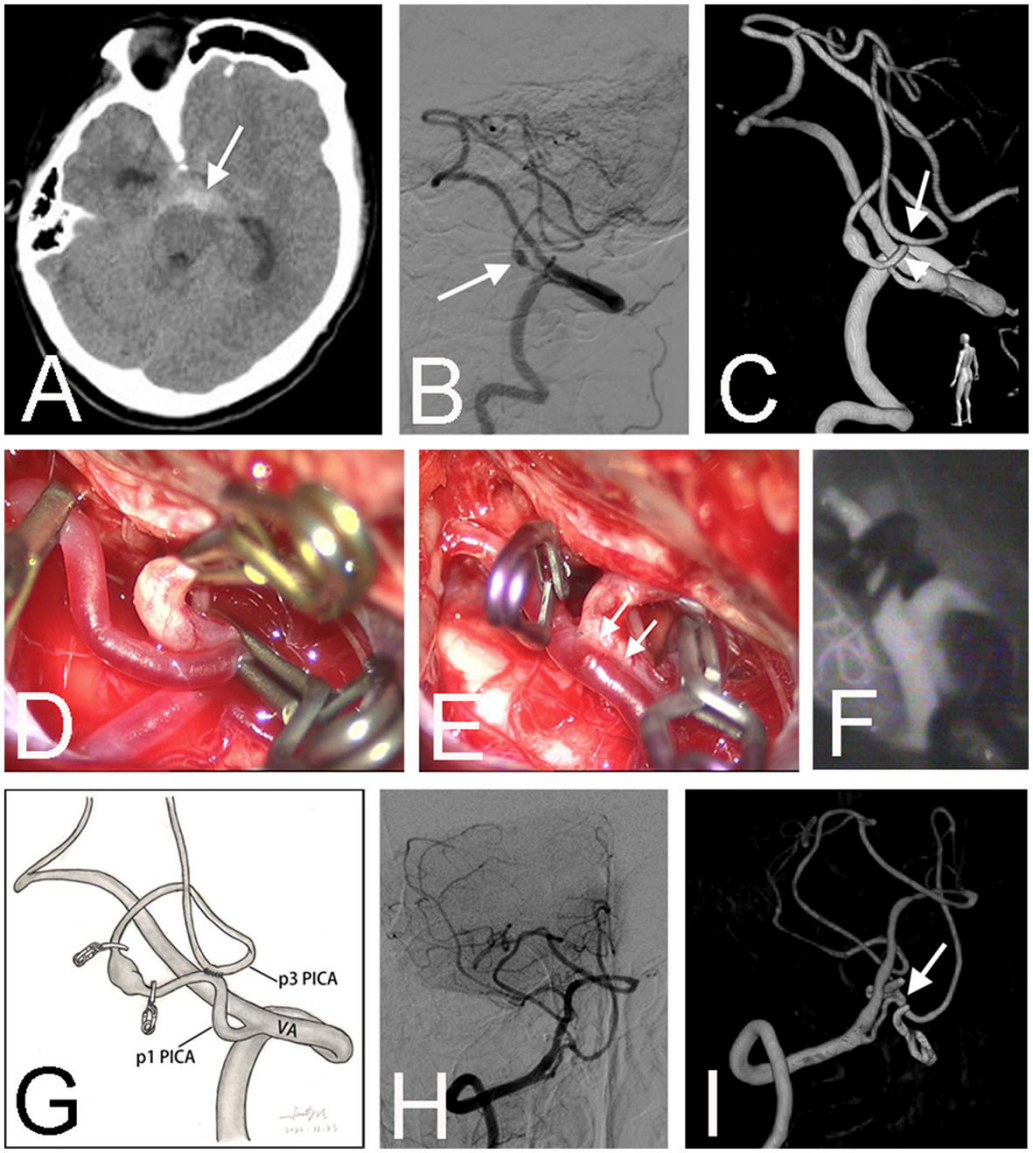
Preoperative CT scan showed a subarachnoid hemorrhage in the interpeduncular cistern (arrow) in a 66-year-old male (Case 6) **(A)**. Right vertebral angiography revealed a fusiform aneurysm (arrow) at the lateral medullary segment (p2) of the right posterior inferior cerebellar artery (PICA) **(B)**. Three-dimensional angiography confirmed the diagnosis and showed that the PICA ran in an unusual pattern, forming a loop between p1 (arrowhead) and p3 (arrow) segments **(C)**. After sufficient dissection, the proximal and distal parts of the loop were easily approximated and temporally occluded by three clips **(D)**. After the *in situ* bypass was completed, the aneurysm was trapped **(E)**. Intraoperative infrared indocyanine green angiography confirmed the patency of the anastomosis **(F)**. The schematic diagram illustrates the treatment strategy for this patient **(G)**. Postoperative angiography demonstrated the obliteration of the fusiform aneurysm **(H)**. Three-dimensional angiography showed the patent anastomosis (arrow) **(I)**.

**Figure 3:**
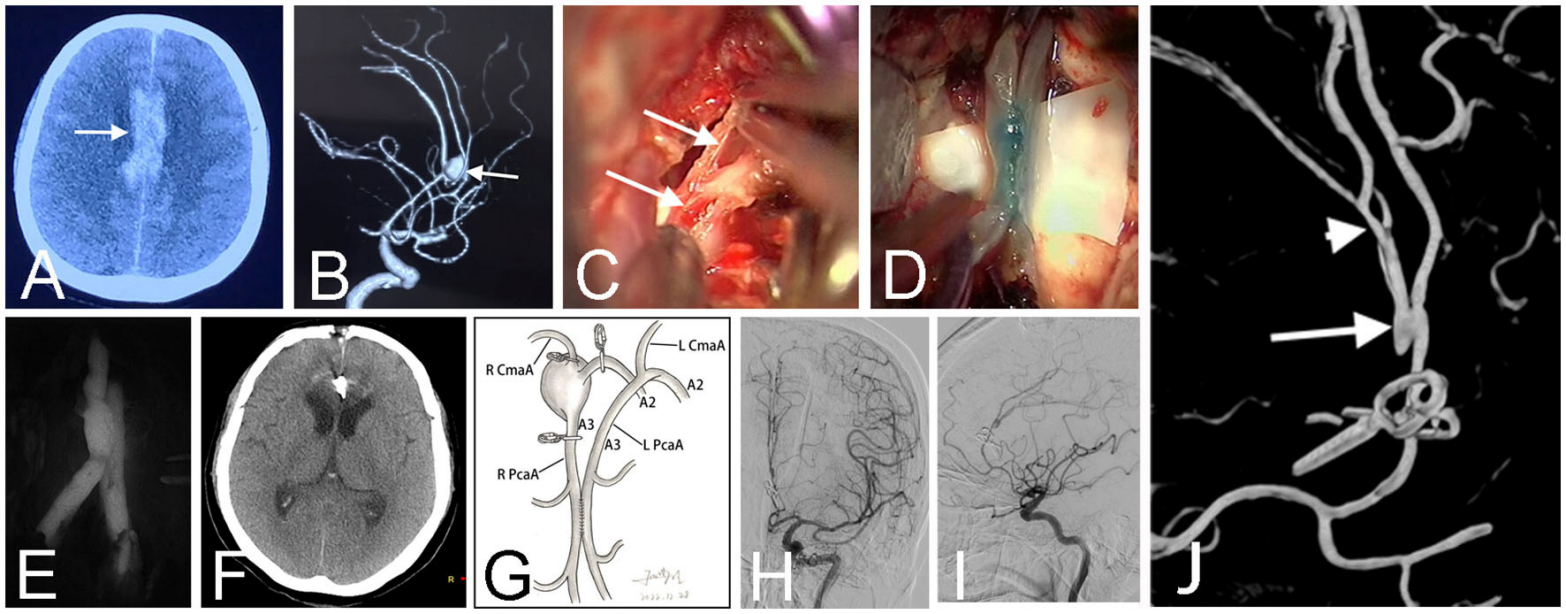
CT scan before the first surgery showed a hematoma (arrow) at the right Sylvian fissure in a 17-year-old girl (Case 12) **(A)**. Pre-operative angiography revealed a fusiform aneurysm (arrow) at the middle cerebral artery **(B)**. The early angiography after the first surgery showed the aneurysm was clamped with multiple parallel straight clips **(C)**. Follow-up angiography demonstrated the aneurysm recurred 1 year after the clipping surgery **(D)**. Intraoperative photograph at the second surgery showed the parallel *in situ* bypasses (arrows) **(E)**. Patency of the two side-to-side anastomosis was confirmed by intraoperative infrared indocyanine green angiography **(F)** and FLOW 800 analysis **(G)**. The schematic diagram illustrated the structure of bypasses and aneurysm trapping while preserving the lenticulostriate artery in this patient **(H)**. Post-operative angiography demonstrated obliteration of the fusiform aneurysm and bold blood flow of distal arteries **(I)**. Three-dimensional angiography showed the patient has two anastomoses (arrows) **(J)**.

In addition to the conventional single simple *in situ* bypass configuration, innovative bypass pattern is efficient in some situations. In our series, we performed a bypass between the proximal and distal parts of the caudal loops within the same PICA (Case 6) (Figure 2). This novel technique had also been reported by Lee and Cho (21) in a patient with a p2 dissecting aneurysm, which they called the “closing omega” technique vividly. In another patient with a MCA recurrent aneurysm after clipping, which characterized an early bifurcation of one of the trifurcated M2 branches, we conducted an ingenious parallel M2-M2 *in situ* bypasses to supply the three branches distal to the aneurysm, which spared the need for a tedious EC-IC interposition bypass or IC-IC reimplantation (Case 12) (Figure 3). Therefore, we firmly believe that with the expanding repertoire of microsurgical techniques and skills, more and more inspired collections of *in situ* bypass structures will be attempted by skilled cerebrovascular specialists.

It should be acknowledged that the present study has some limitations. First, the results of the present study may be biased by the retrospective study nature and the relatively small sample size. Second, this study only reflected the experience and perspective of *in situ* bypasses at a single institution that receives high volumes of patient referrals with complex clinical presentations, so the generalizability of these results is restricted. Third, we did not perform cerebral blood flow evaluation before surgery, and most of our bypass modalities were determined by a multidisciplinary team according to anatomical considerations. This policy had been adopted by most previous reports in the literature, and it also worked in our series. However, the fully preoperative assessment would benefit the surgical complications reduction. We have used CT perfusion to assist in bypass modality selection in certain cases. Last, in our series, the theoretically possible PCA-SCA *in situ* revascularization was not performed, and studies elaborating on this bypass modality and its surgical outcomes were limited. Therefore, future study is necessary to include a larger sample size with a multicenter and prospective design.

## Conclusion

Despite advances in endovascular intervention, the cerebral revascularization technique remains an essential skill for the treatment of complex aneurysms. The *in situ* bypass is one of the most effective techniques to revascularize efferent territory when vital artery sacrifice or occlusion is unavoidable. The configuration of *in situ* bypasses should be carefully tailored to each case, with consideration of variations in the anatomy and pathology of the complex aneurysms.

## Data availability statement

The original contributions presented in the study are included in the article/supplementary material, further inquiries can be directed to the corresponding authors.

## Ethics statement

The studies involving humans were approved by Chinese PLA General Hospital Review Committee. The studies were conducted in accordance with the local legislation and institutional requirements. The participants provided their written informed consent to participate in this study.

## Author contributions

CW and Z-hS: conception and design. H-wW, C-hS, and D-sK: literature search, data extraction, and statistical analysis. H-wW: drafting of the article. ZX and Z-hS: critical revision of the article and study supervision. All authors contributed to the article and approved the submitted version.
